# Pan-genome of *Citrullus* genus highlights the extent of presence/absence variation during domestication and selection

**DOI:** 10.1186/s12864-023-09443-w

**Published:** 2023-06-15

**Authors:** Yang Sun, Dou-Rong Kou, Yan Li, Jiang-Ping Ni, Jing Wang, Yong-Mei Zhang, Qing-Nan Wang, Bin Jiang, Xu Wang, Yue-Xin Sun, Xin-Tong Xu, Xiao-Juan Tan, Yong-Jun Zhang, Xiang-dong Kong

**Affiliations:** 1 Key Laboratory for Conservation and Use of Important Biological Resources of Anhui Province, Anhui Provincial Key Laboratory of Molecular Enzymology and Mechanism of Major Diseases, College of Life Sciences, Anhui Normal University, Wuhu, 241000 Anhui China; 2 Laboratory for Biology of Plant Diseases and Insect Pests, Institute of Plant Protection, Chinese Academy of Agricultural Sciences, Beijing, 100193 China; 3 JiguangGene Biotechnology Co. Ltd, Nanjing, China

**Keywords:** *Citrullus* genus, Pan-genome, Presence or absence variation, RGA, Selection, GWAS

## Abstract

The rich genetic diversity in *Citrullus lanatus* and the other six species in the *Citrullus* genus provides important sources in watermelon breeding. Here, we present the *Citrullus* genus pan-genome based on the 400 *Citrullus* genus resequencing data, showing that 477 Mb contigs and 6249 protein-coding genes were absent in the *Citrullus lanatus* reference genome*.* In the *Citrullus* genus pan-genome, there are a total of 8795 (30.5%) genes that exhibit presence/absence variations (PAVs). Presence/absence variation (PAV) analysis showed that a lot of gene PAV were selected during the domestication and improvement, such as 53 favorable genes and 40 unfavorable genes were identified during the *C. mucosospermus* to *C. lanatus* landrace domestication. We also identified 661 resistance gene analogs (RGAs) in the *Citrullus* genus pan-genome, which contains 90 RGAs (89 variable and 1 core gene) located on the pangenome additional contigs. By gene PAV-based GWAS, 8 gene presence/absence variations were found associated with flesh color. Finally, based on the results of gene PAV selection analysis between watermelon populations with different fruit colors, we identified four non-reference candidate genes associated with carotenoid accumulation, which had a significantly higher frequency in the white flesh. These results will provide an important source for watermelon breeding.

**Supplementary Information:**

The online version contains supplementary material available at 10.1186/s12864-023-09443-w.

## Introduction

Watermelon (*Citrullus lanatus*), which belongs to *Cucurbitaceae* and *Citrullus Schrad*, is an annual or perennial trailing herb. The vine cycle is one year, and most of them are dicotyledons. Watermelon originated from the Sahara Desert in central Africa. It was spread to Egypt and other places around 4000 BC, and then spread to the northern European continent and other regions, as well as spread from the European continent to the east to West Asia, and further spread to China, Japan and other regions of East Asia. Watermelon is an important fruit crop cultivated worldwide, as well as an important economic vegetable crop. The latest classification of watermelon includes 7 species [1]: *Citrullus naudinianus*, *Citrullus ecirrhosus*, *Citrullus rehmii*, *Citrullus colocynthis*, *Citrullus amarus*, *Citrullus mucosospermus* and *Citrullus lanatus*. Watermelon reference (97,103) was released in 2012 [2], which laid a solid foundation for the research of the evolution and breeding of watermelon.

The combination of genes carried by all individuals in the same evolutionary branch in phylogeny constitutes a pan-genome. The pan-genome mainly includes three parts: core genome, dispensable genome and unique genes. Compared with traditional reference genomes, pan-genomes integrate all genes in a species or group, providing new methods and perspectives for species evolution and gene functional research. The pan-genome contains more genetic information which cannot be displayed in the reference genome, such as presence/absence variation (PAV), which refers to a type of structural variation, and present or absent in some accessions as well as play a key role in the phenotype of some accessions. PAV has been widely studied and reported in plant genomes, especially crop genomes. Shen et al. studied the presence or absence of 9 Arabidopsis disease-resistant genes and found that there were 4.3 ± 1.6 genes per plant on average [3]. Springer et al. compared two maize inbred lines B73 and Mo17, and found that at least 180 single-copy genes existed in only one individual [4]. A study of 80 *Arabidopsis* genomefound that 2407 genes (8.9%) were lost in one or more genomes with an average of 444 genes missing per individual [5]. Recently, along with the development of next-generation sequencing technologies and the reduction of sequencing prices, has engendered a genome sequence data deluge in public databases, which provides a better condition for pangenome research. Gao et al. released a tomato pan-genome based on genome sequences of 725 geographically and phylogenetically representative accessions, revealing 4,873 new genes absent from the reference genome [6]. Golicz et al. assembled and analyzed a *Brassica oleracea* pangenome with 10 *B. oleracea* varieties and identified 61,379 genes on pangenome [7]. Li et al. assembled a soybean (*Glycine soja*) genome based on 7 wild type soybeans, and identified genes with copy number variation or which exist in some specific individuals, and some of them regulate certain agricultural traits such as biological resistance [8].

Resistance gene analog (RGA) is a class of genes that have conserved domains and motifs [9], it includes PRR and R genes. Most of the characterized PRR are surface-localized receptor-like protein kinase (RLK) or membrane-associated receptor-like protein (RLP) [10–12]. There are two groups of PRRs: surface-localized receptor-like protein kinases (RLKs) [13] and membrane associated receptor-like proteins (RLPs). RLKs and RLPs are important for plant disease resistance [14]. Most R genes are composed of nucleotide binding domain (NB) and leucine-rich repeat (LRR) domain, which is usually called (NB-LRR) R gene or NLR [15].

Over the course of more than 4000 years of domestication and improvement, watermelon has undergone significant changes in fruit quality-related traits compared to its wild ancestor. Guo et al. reported the whole-genome resequencing of 414 representing accessions of Citrullus genus [16]. They had revealed some genetic loci associated with watermelon fruit size that have been under selection during speciation, domestication, and improvement. The present study aims to explore domestication, evaluate breeding history, and gain a more complete understanding of genetic elements by constructing a pan-genome that includes both cultivated watermelon and its wild ancestor. In the present study, resequencing data of 400 accessions were downloaded and then used to de novo assemble the *Citrullus genus* pangenome. As a result, 477 Mb new sequences and 6249 new protein-coding genes were obtained. By comparative analyses, 180, 5 and 53 favorable genes, 29, 33 and 40 unfavorable genes were identified in CM to CL-cultivar, CL-landrace to CL-cultivar and CM to CL-landrace domestication and improvement. Resistance is an important trait of watermelon, so we identified resistance gene analogs (RGAs) in *Citrullus genus* pangenome and 661 RGA candidates were obtained. Furthermore, we linked RGA with known QTL. These results provide a valuable genomic resource and bring a new sight for crop breeding in *Citrullus genus*.

## Results

### Citrullus genus pan-genome construction

The 400 watermelon accessions analyzed in the present study represent all extant species in the *Citrullus* genus (*Citrullus amarus, Citrullus colocynthis, Citrullus ecirrhosus, Citrullus lanatus, Citrullus mucosospermus, Citrullus naudinianus* and *Citrullus rehmii*). Clean data of 400 accessions were do novo assembled, after removing the contigs shorter than 500 bp, a total of 95 Gb contigs were retained. All the contigs were aligned to the reference to identify non-reference sequences. After merging the fully unaligned contigs and partially unaligned contigs sequences, cd-hit was used to remove redundant and obtained 964.33 M sequences. By removing the non-Eukaryota and non-Viridiplantae sequences and performing multiple rounds of all vs all alignment to remove redundancy, 477 Mb clean non-reference sequences were obtained finally. Among these contigs, 413 Mb sequences came from other six species of *Citrullus* genus, and 64 Mb sequences came from *Citrullus lanatus*. A total of 6249 PCgenes (protein-coding genes) were predicted in the non-reference genome. The melon reference genome contains 22,596 PCgenes, so there are 28,845 genes in the pangenome.

**Fig. 1:**
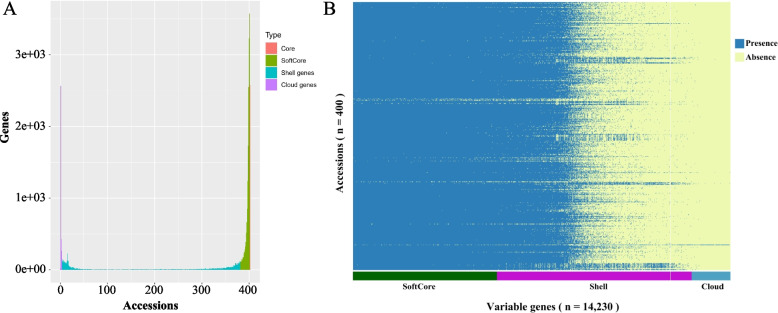
**A,** Composition of the watermelon pan-genome. **B**, Heatmap of variable genes present or absent in 400 accessions

By calculating the read coverage of each gene in the *Citrullus* genus pan-genome, we identified the gene present and absent variations in each sample. Like the classification criteria described by Gao et al. [6], a total of 20,050 core and softcore genes, 7,341 shell genes and 1,454 cloud genes (Fig. 1A) were identified. Core and softcore genes exist in all accessions or nearly all accessions, while shell genes and cloud genes lost in many different accessions (Fig. 1B).

### Selection of gene PAVs during watermelon breeding

**Fig. 2:**
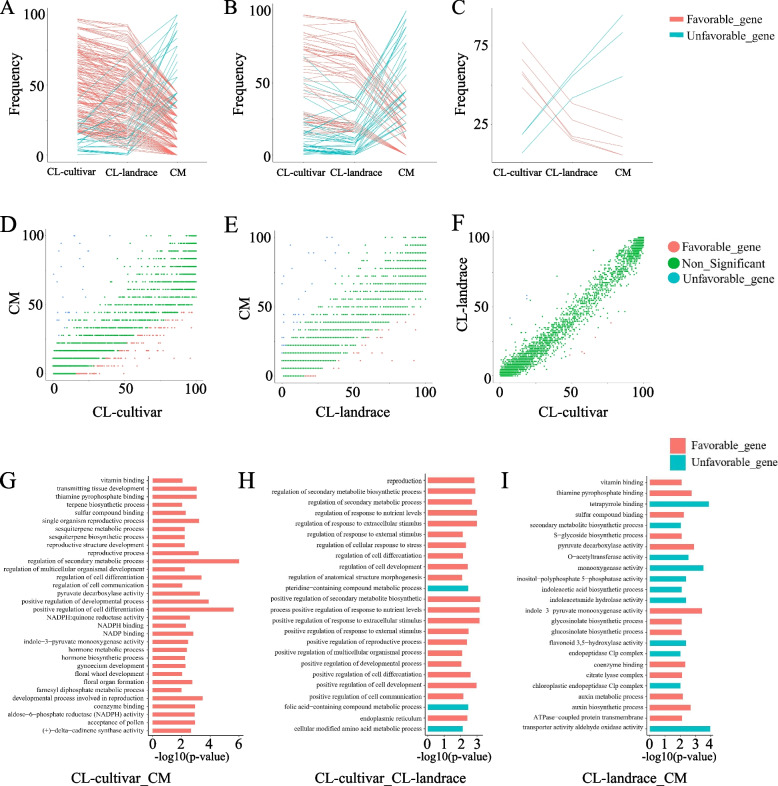
**A**-**C**. Scatter plots showing genes frequencies in CL-landrace vs CM, CL-cultivar vs CM and CL-cultivar vs CL-landrace. **D**-**F**. Line plot showing the trends of favorable genes and unfavorable genes (from left to right are CL-cultivar vs CM CL-landrace vs CM, and CL-cultivar vs CL-landrace respectively) during the process of domestication and improvement.. **G**-**I** GO enrichment display of favorable and unfavorable genes in CL-cultivar vs CM, CL-cultivar vs CL-landrace and CL-landrace vs CM

To identify gene PAVs under selection during the domestication and improvement process of watermelon. We conduct gene PAV selection analysis between *C. mucosospermus* (CM), *C. lanatus* landrace (CL-landrace) and *C. lanatus* cultivars (CL-cultivar). In total, we identified 180, 5 and 53 favorable genes as well as 29, 3 and 40 unfavorable genes in groups of CL-cultivar vs CM, CL-cultivar vs CL-landrace and CL-landrace vs CM respectively (Fig. 2A-F, Table S3-5). These results indicated that many genes were disproportionally lost during both domestication and improvement. Furthermore, there were 209 and 93 selected genes in CL-cultivar vs CM and CL-landrace vs CM, but there were only 8 present genes were selected in CL-cultivar vs CL-landrace. Moreover, GO enrichment analysis was performed in the favorable and unfavorable genes of three comparison groups (Fig. 2 G-I). Favorable genes enriched in positive regulation of response to nutrient levels, terpene biosynthetic process and secondary metabolite biosynthetic process which may relate to the flavor of fruit and stress response indicating that fruit-quality-related genes [16] and stress response-related genes [17] were selected in domestication and improvement.

### Distribution and PAV selection of RGA candidates

**Fig. 3:**
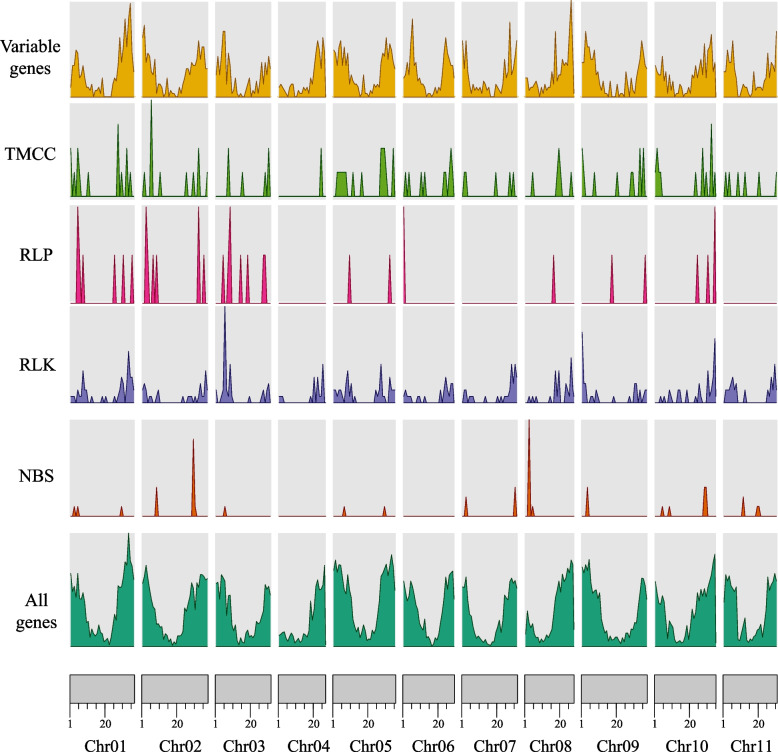
The density of genes compared with the density of NBS, RLK, RLP as well as variable genes

A total of 661 RGAs were identified in the *Citrullus lanatus* genome (Fig. 3, Table 1). RLK comprising 417 genes was the largest class of resistance gene candidates, followed by TM-CC genes (120; Table 1). Of these 661 genes, 555 (84.0%) were core genes (softcore and core genes) and 106 (16.0%) were variable genes (cloud and shell genes). A total of 571 RGAs were on the reference (554 core and 17 variable), and 90 RGAs (1 core and 89 variable) were identified on the pangenome additional contigs. The density of different types of RGA genes per pseudomolecule was roughly similar to all pseudomolecules (RGA candidates per Mb ranging from 0.8 on pseudomolecule 04 to 2.0 on 01, average 1.57 per Mb), but was different between different types (TM-CC genes per Mb ranging from 0.47 on pseudomolecule 05 to 0.07 on 11; RLK genes per Mb ranging from 1.16 on pseudomolecule 11 to 0.73 on 04; There were no RLP genes located on pseudomolecule 04, 07 and 11, and the 0.25 per Mb was the highest density of RLP on pseudomolecule 03; There was also no NBS genes on pseudomolecule 04 and 06, and the density on pseudomolecule 03 up to 0.39). Furthermore, the additional contigs harbored more NBS-LRR and RLK than RLP candidate genes (31, 29, and 10 respectively).In the present study, we identified 49 NBS-LRR genes on the reference genome, which number was roughly similar to the number of NBS-LRR genes identified in a previous study [2].

**Table 1:** The number of different RGA candidates and subfamilies found on the reference genomes, pangenome additional contigs and reference genome unplaced contigs. The numbers in parentheses represent the number of variable genes (cloud and shell genes) and core genes (softcore and core genes), respectively

RGAs	Reference	Pangenome additional contigs	Pangenome
CN	2 (0, 2)	3 (3, 0)	5 (3, 2)
CNL	10 (0, 10)	2 (2, 0)	12 (2, 10)
NBS	6 (0, 6)	9 (9, 0)	15 (9, 6)
NL	10 (0, 10)	6 (6, 0)	16 (6, 10)
RLK	388 (9, 379)	29 (28, 1)	417 (37, 380)
RLP	34 (2, 32)	10 (10, 0)	44 (12, 32)
TMCC	100 (2, 98)	20 (20, 0)	120 (22, 98)
TN	2 (0, 2)	0 (0, 0)	2 (0, 2)
TNL	7 (1, 6)	3 (3, 0)	10 (4, 6)
TX	7 (3, 4)	8 (8, 0)	15 (11, 4)
OTHER	5 (0, 5)	0 (0, 0)	5 (0, 5)
Total	571 (17, 554)	90 (89, 1)	661 (106, 555)

Of the RGA candidate genes, 555 were present in most lines (softcore and core), and 106 were variable genes (cloud and shell). NBS-LRR showed the highest number of variance (43.8.0%), followed by RLP (27.3%), while RLK showed the lowest percentage of variable genes (8.9%). The high percentage of variable genes of NBS-LRR could mean that these genes are pseudogenes that the genome can afford to lose without consequences in the form of lost resistance.

Different types of RGA candidates were compared within different accessions based on PAV results (Figure S1). RGA candidates of different types showed the same present and absent trends in different types of accessions. The present NBS candidates account for the lowest proportion compared to other types of RGAs (NBS candidates present in more than 90% of samples accounted for 60%), followed by RLP, which present in more than 90% of samples accounted for 77.2%. The presence of RLKs present over 90% of samples was 94.0%, which was the largest proportion among all RGAs. These results revealed that NBS candidates were the most changeable subfamily and RLKs were the most stable subfamily among RGAs.

### Linking known QTL and R-genes

**Fig. 4:**
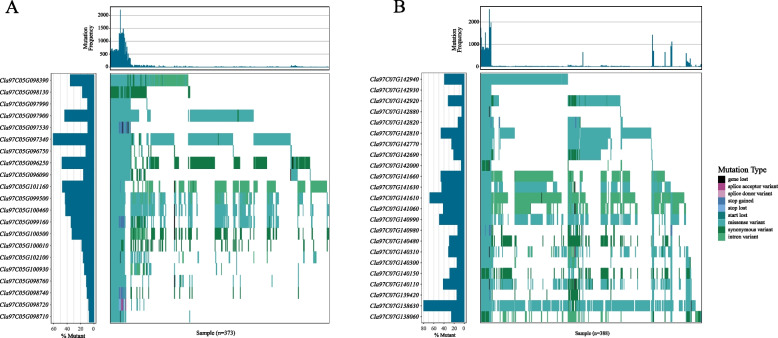
Waterfall plot of the gummy stem blight resistance-linked QTL ClGSB5.1 (**A**) and ClGSB7.1 **B**

The RGA candidate positions were compared with three known quantitative trait loci (QTL) for gummy stem blight [18] (*ClGSB3.1* on chr3, *ClGSB5.1* on chr5 and *ClGSB7.1* on chr7 respectively). These QTLs cover 18.5 Mb on three chromosomes with an average of 6.2 Mb. Among the three QTLs, only one RGA gene in the *ClGSB3.1* was mutated in 157 varieties, and the mutation types were start lost, missense variant, synonymous variant and intron variant. On the whole, there were a large number of missense variants in the *ClGSB5.1* and *ClGSB7.1* (Fig. 4A-B), indicating that these two QTLs are under positive selection pressure. Among all the mutated genes in *ClGSB5.1*, *Cla97C05G097340* had the highest mutation rate, indicating that *Cla97C05G097340* was under the most selective pressure among all varieties, and the mutation type was mainly a missense variant. *Cla97C05G098710* has the lowest mutation frequency among all the mutated genes, indicating that among all the mutated genes, *Cla97C05G098710* was under relatively lower selection pressure. In the *ClGSB7.1*, the mutation rate of *Cla97C07G138630* was the highest, indicating that it was under higher selection pressure, and the type of mutation was mainly missense variant.

### Gene PAV-based GWAS

**Fig. 5:**
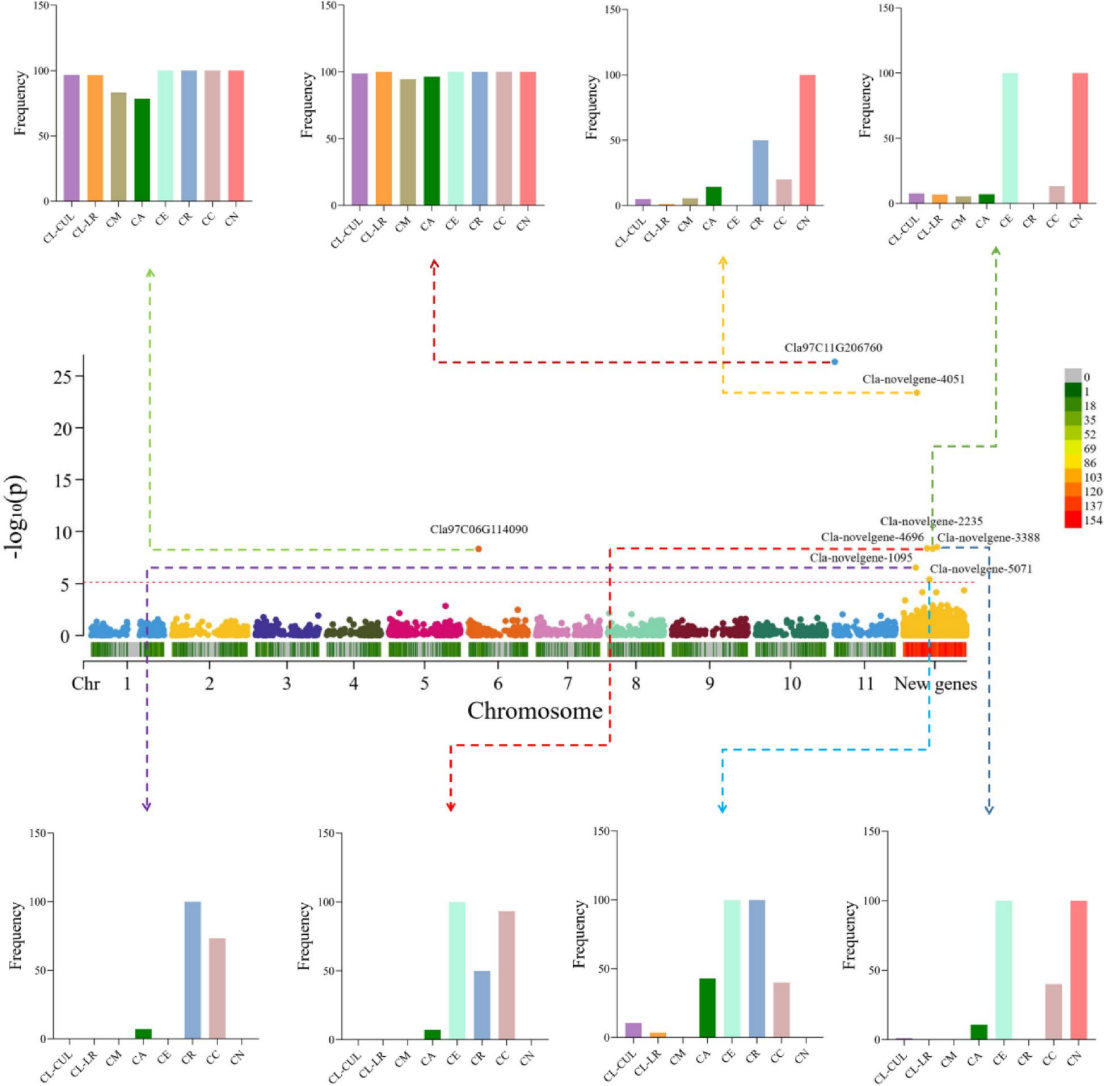
Gene PAV-based GWAS of flesh color. The histogram represents the frequency of genes in different groups. The meaning of the abbreviation is as follows: CA, C. amarus; CC, C. colocynthis; CE, C. ecirrhosus; CL_CUL, C. lanatus cultivar; CL_LR, C. lanatus landrace; CM, C. mucosospermus; CN, C. naudinianus; CR, C. rehmii

Presence/absence variation (PAV) is a major class of genome structure variation (SV), and gene PAV is a class of PAV. Gene PAV can also cause the change of phenotype. In a present study, shell genes were used to conduct the gene PAV-based GWAS, which may analyze the genes located in the reference genome and additional pangenome contigs. We identified 8 gene PAVs associated with flesh color (Fig. 5), 6 of which were on the additional pangenome contigs. Among them, Cla-novelgene -4696 and Cla-novelgene -3388 are genes containing the lipoxygenase domain and they were lost in CL-CUL, CL-LR, CM and CR populations, while there are high frequencies in CE and CN. Cla-novelgene-1095 is a thaumatin domain containing gene that has a high frequency in CR and CC and is absent or has a very low frequency in other populations. The Cla97C11G206760 gene located on the reference gene is a gene containing the adh_short (PF00106) domain, which has a high frequency in all varieties. Three other non-reference novel genes (Cla-novelgene4051, Cla-novelgene-2235 and Cla-novelgene-5071) contain the A_thal_3526 (pfam09713), Myb_DNA-binding and probable lipid transfer domain.

### PAV selection and expression analysis of carotenoid-related genes

**Fig. 6:**
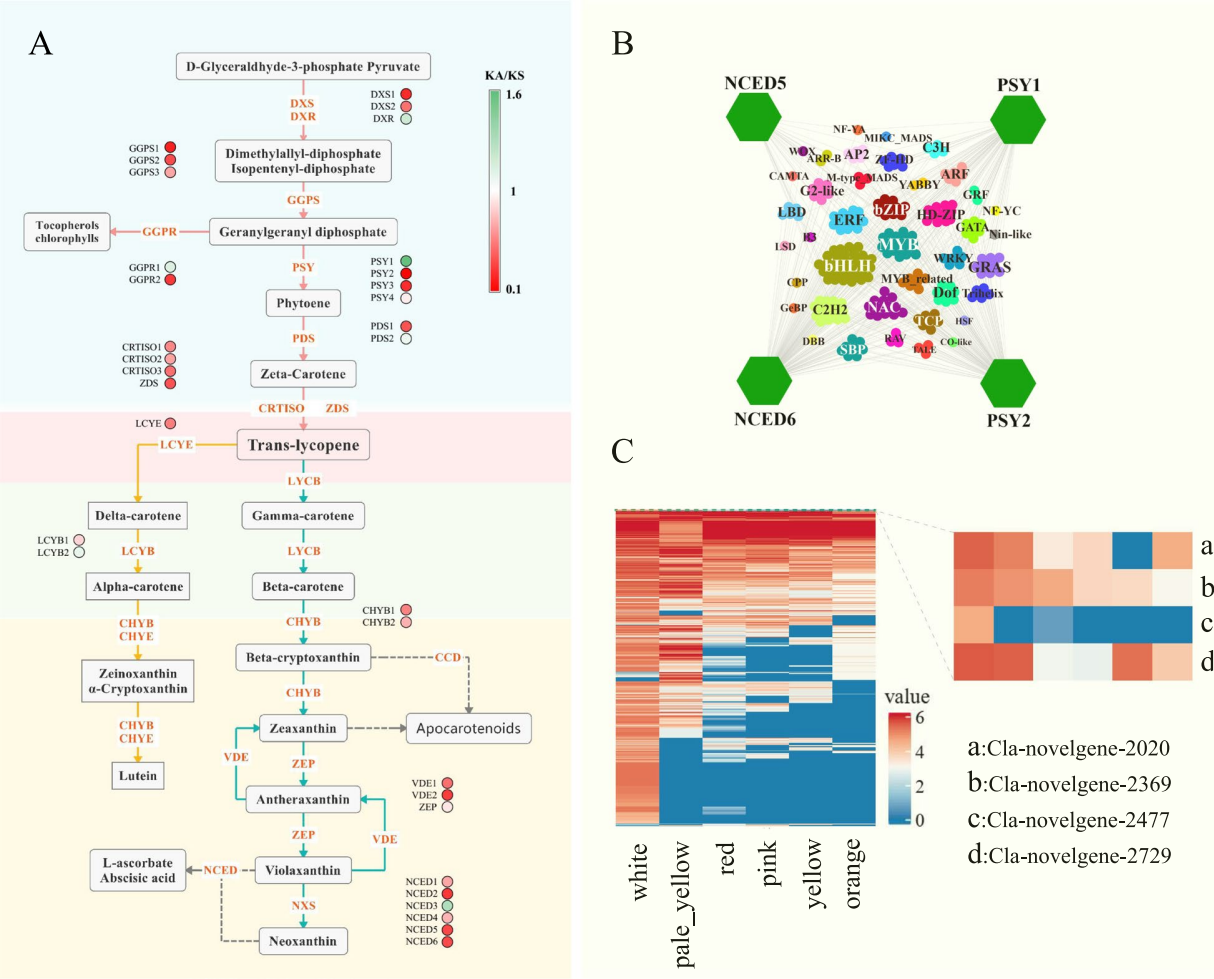
**A**, The Ka/Ks value of genes involved in carotenoid biosynthesis pathway. **B**, Network of transcription factor regulation of carotenoid biosynthesis-related genes. The color-filled hexagons represent the differentially expressed carotenoid biosynthesis-related genes. Correlations between TFs and carotenoid biosynthesis-related genes are calculated by Pearson’s correlation test (*P* ≤ 1e-6). **C**, The heatmap of the genes under PAV selection between different flesh color watermelon populations

Carotenoid accumulation is closely related to the color of watermelon flesh, and in this study, PAV, SNP and expression of genes in the carotenoid synthesis pathway were analyzed. All genes involved in the carotenoid synthesis pathway were core or softcore genes, indicating that these gene PAVs were not under selection. However, the ka/ks values calculated based on population SNPs showed that some carotenoid-related genes were positively selected (Fig. 6A). Ka/ks values of most carotenoid-related genes were less than 1, indicating that these genes were under purifying selection. A few genes, such as PSY1, NCED3 and DXR, had ka/ks values greater than 1 indicating that they were under positive selection.

A total of 15,122 differentially expressed genes were identified between different varieties or between different periods of the same variety. Based on these DEGs, WGCNA-based co-expression analysis was performed, and a total of 19 gene expression modules were identified. The results showed that four carotenoid-related genes were present in one expression module. A large number of transcription factors in these modules may regulate these carotenoid-related genes. BHLH, MYB and NAC are transcription factors that may regulate NCED5, NCED6, PSY1 and PSY2 (Fig. 6B).

The resequencing population of watermelon was divided into five different flesh colors. A total of 3344 genes that were subject to gene PAV selection between the different color populations (Fig. 6C) were identified by gene PAV selection analysis between these populations. Interestingly, four genes related to carotenoids were under selection, two of these four genes containing the pfam04116 functional domain, and two others were genes containing the pfam05834 domain and carotenoid cleavage dioxygenase 7, respectively.

## Discussion

The research of Guo et al. [19] revealed the evolution history of watermelon. In the process of domestication and improvement, different loci on the genome that affect the characters of watermelon have been selected. These results were based on the updated watermelon genome (97,103), and the selection signals are analyzed by identifying the SNP of the genome. However, many new genes are produced in the process of evolution and domestication. In this study, the pan-genome based on 400 watermelon and other six watermelon species contains 477 Mb new sequence, which is larger than the reference genome size of watermelon. 413 Mb sequences came from the other six species of Citrullus genus, and 64 Mb sequences came from *Citrullus lanatus*. It shows that the interspecific difference of watermelon is large, while the intraspecific difference of *Citrullus lanatus* is small. The interspecific difference of watermelon is smaller than radish, because the pan-genome of radish constructed by Xiaohui Zhang et al. contains 150,757 non-redundant genes [20], which is three times that of a single genome. At the same time, the gene diversity of watermelon is higher than *Capsicum*. The pangenomic sequence of *Capsicum* has a total of 956.43 Mb non-reference, accounting for only 28.4% of the reference genome [21].

Pan-genome analysis allows us to understand the phenomenon of gene diversity in the process of speciation, domestication and improvement from the perspective of gene loss. The Fisher's exact test was performed based on the gene PAV matrix and the threshold was set to *P* < 0.001, which is the standard used in tomato, cotton and chicken genomes [6, 22, 23]. By distinguishing the favorable genes from the unfavorable genes, the corresponding genes in wild species or adjacent species can be found and applied to breeding. CM is the species closest to the CL relationship, although it has been artificially selected, some characteristics of its flesh have not been artificially selected [19]. Our gene PAV selection analysis results showed that CL-landrace and the genes with different gene frequencies between CL-cultivar and CM were enriched in many GO terms. For example, in the comparison of gene frequency between CL-cultivar and CM, most of the significant genes have high frequency (favorable genes) in CL-cultivar. Enrichment analysis shows that favorable genes are enriched in sesquiterpene biosynthetic process, which may be associated with the processes that convert the ovary of the female flower into a fruit [24]. In the PAV selection analysis results of CL-landrace vs CM, some GO terms related to plant stress resistance were enriched in both unfavorable genes and favorable genes, for example, favorable genes were enriched in sulfur compound binding possibly associated with cadmium stress [25], unfavorable genes enriched in flavonoid 3',5'-hydroxylase activity may be associated with oxidative stress [26]. The PAV of these genes provides a better understanding of the effects of artificial selection on the genome and the possible relationship between gene PAV selection and phenotypes.

Resistance gene analogs (RGAs), which contain genes such as NBS-encoding proteins, receptor-like protein kinases (RLKs) and receptor-like proteins (RLPs), are R-genes containing specific functional domains. Identification and analysis of RGAs are important for plant studying. In watermelon, some previous studies have identified and analyzed NBS-Encoding and other genes [2, 27] However, these studies had not identified all RGA genes, and they were based on the reference genome. So, the results cannot cover the gene catalogs that are not present in the reference genome. In the RGA studies of *Brassica oleracea* and *Brassica napus*, a large number of RGAs absent in the reference genome were identified [28, 29], and their variation in the population was analyzed, which provided valuable information for resistance breeding. In this study, based on the watermelon pan-genome, 661 RGAs were identified, of which 90 RGAs were on the pangenome additional contigs. Most of these non-reference RGAs are variable genes (only 1 RGA is core gene). Among the 571 RGAs in the reference genome, 17 are variable genes, which indicate that some resistance genes have undergone selection events among different species of watermelon. The number of NBS-LRR genes in the watermelon genome is similar to cucumber (61) [30] and papaya (35) [31] but is considerably fewer than that in rice (600) [32] and apple (575) [33].There were more RLK than NBS-LRR and RLP genes probably caused by the diverse functions of genes in different classes. RLK genes have been reported that involved in a variety of regulatory progress such as interactions with symbionts, self-incompatibility and regulation of growth processes in response to hormones [34] so they are not necessary for the progress of resistance. However, other RGA genes like NBS-LRR genes mainly focus on resistance [35]. We found that the largest class of RGA candidates was RLKs, which is consistent with other plants such as *B. oleracea*, wild strawberry and cotton [28, 36, 37]. Combined with the resistance traits of species or varieties, it can provide very valuable information for watermelon resistance breeding in the future.

The gene PAV is a structural variation that can cause phenotypic changes. Pangenomic study of rape and pigeonpea by gene PAV-based GWAS found that PAV-GWAS could identify the candidate genes that SNP-GWAS could not identify. This can provide new insights for the understanding of the genetic mechanism of crop traits, and may find some new candidate genes associated with traits. For example, in the pangenomic study of chicken, researchers found that a PAV in the IGF2BP1 promoter region was associated with chicken growth traits, including claw weight (CW1), the ratio of claw weight to body weight (CR), double pinion weight (DPW), and semi-evisceration weight (SEW) through PAV-GWAS and PAV selection analysis [23]. In the present study, gene PAV-based GWAS was performed using the flesh color phenotypic data of watermelon, and 8 candidate genes were found to be significantly related to the phenotype, which was different from the candidate genes identified by Guo et al., based on SNP-GWAS [19]. Cla97C11G206760 is a gene containing a short chain dehydrogenase domian. The cytosolic short-chain dehydrogenase with this domain can convert xanthoxin to ABA-aldehyde during ABA synthesis, which may be associated with the orange color of melon flesh [38]. The relationship of this gene PAV with the flesh color of watermelon needs further study. Two non-reference genes, Cla-novelgene-3388 and Cla-novelgene-4696, both belong to lipoxygenase, a gene family that may be highly expressed during fruit ripening induced by ABA, which are associated with fruit color formation and ethylene production, etc. [39]. However, whether these two genes affect the flesh color of watermelon in some indirect way needs further study. The combination of the functions of these genes and their presence/absence variation in the genome is worthy of further study.

Some gene families may undergo functional differentiation as they undergo gene duplication and family expansion [40]. So we found that genes of the same family are not subjected to the same selection pressure, for example, PSY1 during carotenoid synthesis is subjected to positive selection pressure, while PSY2 and PSY3 are subjected to a stronger purifying selection. Flesh color is an important trait in watermelon, and gene PAV selection analysis in different color watermelon populations can be effective in understanding the role of selection on gene PAV during flesh color-based domestication. In this study, we found that most of the genes subjected to gene PAV selection among five different color watermelon populations had a high frequency in the white flesh population. Based on their functional annotation, four genes associated with carotenoid accumulation were identified. These four genes were novel genes annotated on the non-reference contigs. The superfamily with the pfam04116 domain includes fatty acid and carotene hydroxylases and sterol desaturases. It may play a negative role in carotenoid accumulation, such as in a potato study that found that β-carotene hydroxylase silencing increased total and β-carotene levels in potato tubers [41]. The gene family containing the pfam05834 domain contains lycopene beta and epsilon cyclase proteins. Interestingly, CRISPR-based editing of the LCYε gene resulting in point mutations/premature termination of the LCYε protein was found to result in a sixfold increase in β-carotene content and a decrease in α-carotene and lutein content [42]. There is also a carotenoid cleavage dioxygenase with a higher frequency in white watermelon associated with white fruit flesh [43]. These results suggest that genes with a high frequency in white watermelon, especially the four genes potentially associated with carotenoid accumulation, are candidates genes related to watermelon flesh color. And these four genes are all absented in the reference genome, illustrating that pan-genome studies can help to identify more candidate genes.

## Material and method

### Genome sequences of Citrullus genus

Whole genome resequencing data of 400 cultivated and wild watermelon accessions (Table S1) were downloaded from the NCBI Sequence Read Archive (SRA) through the SRA accession numbers SRP188834 [19], including 337 *Citrullus lanatus* accessions, 28 *Citrullus amarus* accessions, 15 *Citrullus colocynthis* accessions, 1 *Citrullus ecirrhosus* accessions, 18 *Citrullus mucosospermus* accessions, 1 *Citrullus naudinianus* accessions and 2 *Citrullus rehmii* accessions. SRA Toolkit's Fastq-dump (http://www.ncbi.nlm.nih.gov/Traces/sra/sra.cgi?view=toolkit_doc&f=fastq-dump) was used to convert SRA into fastq format. Fastp was used to remove adapters and low-quality sequences in the raw data [44].

### Citrullus genus Pan-genome construction

The pan-genome was constructed according to the method described by Gao Lei et al. [6]. Each sample was de novo assembled using Megahit [45]. After de novo assembly, the contigs shorter than 500 bp were removed. Then we used the nucmer of Mummer software package [46] to align the remaining contigs with the watermelon reference genome and organelle genome. Watermelon reference genome‘97,103’ (version 2) and genome annotation files were downloaded from the Cucurbit Genomics Database ([19]. The contigs contained sequence identity with watermelon reference genome exceeding 90% and the length exceeding 300 bp were considered to be a reliable alignment. Contigs that had no reliable alignment were considered to be fully unaligned contigs. Among the sequence aligned with the reference genome, these regions which longer than 500 bp with identity of less than 90% were considered to be partly unaligned contigs.

Cd-hit-est [47] was used to remove redundant sequences after merging partially unaligned contigs and fully unaligned contigs. To further remove redundancy, blastn and numcer were used to perform all vs all alignment, and in-house perl scripts were used to filter the aligned results. 90% of regions with 90% sequence identity were set as the threshold for further removing redundant sequences. Finally, blastn and NT were used to aligned non-redundant sequences, and sequences not belonging to Eukaryota or sequences belonging to Eukaryota but not belonging to Viridiplantae were removed. To ensure that these new sequences did not exist in the reference genome, the sequences obtained in the previous step were aligned to the reference genome using blastn once again, and similar sequences were filtered using nucmer with the same criteria as described above. The pan-genome was finally obtained by combining the new sequence with the melon reference.

### Pan-genome annotation

RepeatModeler [48] was used to de novo build a repeat sequence database. After removing contaminated sequences, we used RepeatMasker [49, 50] to annotate repeated regions of the non-reference sequence. We used tandem repeats finder (TRF) [51] to annotate the tandem repeats. At the protein level, we used RepeatProteinMask (an application within the RepeatMasker package) to search the repeated sequences in the TE protein database.

The maker2 [52] software was used to predict the gene structure of the genome based on the repeat sequences masked contigs. We used Augustus [53] for de novo gene prediction, and *Citrullus lanatus* was used as model which was trained by the watermelon reference annotation. RNA-seq data of 90 accessions (Table S2) were used as transcription evidence. Fastp was used to remove adaptors and low-quality sequences in raw data of RNA-seq [44]. We used Hisat2 [54] to map reads to non-reference contigs, and samtools was used to extract reads that can be aligned with non-reference sequences [55]. Trinity [56] was used to de novo assemble reads which mapped to non-reference sequences in each sample. After merging the 166 assembled transcript sequences, we used cd-hit-est [47] to remove redundancy (use default parameters). Finally, the annotation of the non-reference sequence was obtained through maker2. We removed the genes which overlapped 50% with the repeat annotations. Furthermore, Interproscan [57] was used to annotate gene sequences and genes annotated with interpro domains remained.

The predicted gene sequences were compared with the NT database using blastn, and compared with NR database, uniport database and swissport database using blastx. At the same time, GO [58, 59] and KEGG annotations ([60–62] were obtained through the correspondence between the databases.

### Identification and analysis of gene PAV

After the reference genome and non-reference sequence were merged as pangenome, we used bowtie2 [63] to align 400 resequencing data to the pangenome. SGSGeneLoss was used to determine the presence and absence of genes in each sample [64]. We used the parameters: minCov = 2 and lostCutoff = 0.2, which means that a gene was treated as lost when 20% of its region has less than 2 reads. This criterion that has been applied in pan-genomic studies in cotton, tomato, etc. [6, 22].According to described by Gao Lei et al. [6], the genes that exist in all accessions were defined as core genes, genes exist in 99%-100% of accessions were defined as soft-core genes, and genes exist in 1%-99% samples were defined as shell genes. Genes presented less than 1% accessions were defined as cloud genes.

For further identifying the selected gene PAVs during the domestication and improvement, we calculated the frequency of each gene (*Citrullus lanatus* landrace vs *Citrullus mucosospermus* and *Citrullus lanatus* landrace vs *Citrullus mucosospermus*) and improvement (*Citrullus lanatus* cultivar vs *Citrullus lanatus* landrace). Fisher's exact test was used to test the significance of the difference in gene frequency between groups, and the Benjamini and Hochberg method was used to obtain FDR from the corrected p-value. For each comparison, genes with significantly different frequencies ( |log2 (frequency fold change)|> 2 and FDR < 0.001) between the two groups were identified as selected gene PAVs [6, 65]. In addition, we performed GO and KEGG enrichment analysis on selected genes. We defined genes as favorable genes which had higher frequencies in CL-cultivar than CM and CL-landrace, or had higher frequencies in CL-landrace than CM. On the contrary, genes were defined as unfavorable genes which had frequencies lower in CM than CL-landrace and CL-cultivar or had frequencies lower in CL-landrace than CL-cultivar.

### Gene PAV-based GWAS

We downloaded the phenotype (color) of watermelon from Supplementary Table 16 in the study of Guo et al. [19]. The shell genes were performed PAV-GWAS, and the gene presence and absence were used as the genotype. GWAS was conducted using FarmCPU (default parameters) [66] in rMVP [67], and the significance threshold was set to 8.1e-6 (0.05/6161). Meanwhile, 10 SNP-based principal components were used as covariates.

### Identification and analysis of resistance genes

The RGAugury pipeline [68] was used to identify RGA (resistance gene analogs) candidates in the pan-genome of watermelon. RGAs include NBS-LRR, RLP, RLK and TMCC, and can be further divided into 12 subfamilies. According to the results of PAV analysis, these resistance genes are divided into two groups (core genes and variable genes). SNP data of the watermelon population were downloaded(ftp://cucurbitgenomics.org/pub/cucurbit/reseq/watermelon/v2/) and annotated with Variant Effect Predictor (VEP) v99 [69]. The raw genome intervals (based on Charleston Gray watermelon genome) were converted to genome interval of 97,103 v2 genome. Overlapping of genes and QTL regions were determined using bedtools v2.16.2 intersect [70]. Known gummy stem blight resistance-linked QTL were collected from the study of Gimode [18]. Waterfall plots with SNP and PAV information were drawn using GenVisR v1.11.3 [71].

### Transcriptome analysis of watermelon with different flesh colors

RNA-seq data of 45 samples during the fruit development of cultivated watermelon with five different flesh colors were downloaded for NCBI SRA database (PRJNA644468) [72]. Adapters and low-quality sequences in the raw RNA-seq data were removed by Fastp. Clean data were mapped to the‘97,103’ (version 2) genome by hisat2 [73]. The reads counts of each gene were calculated using featureCounts [74]. DESeq2 was used to identify differentially expressed genes in watermelon between different flesh colors and between different developmental periods [75]. All differentially expressed genes were used for WGCNA analysis and the expression of these genes was calculated using FPKM values [76]. Transcription factors with co-expression relationships with differentially expressed carotenoid synthesis pathway-related genes were obtained by the module of WGCNA analysis. The interaction of transcription factors with genes related to the carotenoid synthesis pathway wasvisualized by Cytoscape [77].

### Analysis of genes related to carotenoid synthesis

The CDS sequences of all genes in the 97,103 v2 genome were used for blastn-based alignment with the CDS sequences of genes involved in the carotenoid synthesis pathway. Based on the SNP information of the watermelon population, kaks.py in selectionTools (https://github.com/MerrimanLab/selectionTools) was used to calculate the ka/ks values of each carotenoid synthesis-related gene [78].

## Supplementary Information

**Additional file 1: Figure S1.** Heatmap of NBS (A), RLK (B), RLP (C), TM-CC (D) and all RGAs (E) presence and absence variations in different accessions.

**Additional file 2: Table S1.** List of samples used for Citrullus genus pan-genome construction.

**Additional file 3: Table S2.** List of the SRA number of RNA-seq data used for gene structure annotation.

**Additional file 4: Table S3.** The result of PAV selection analysis between CL-cultivar and CM.

**Additional file 5: Table S4.** The result of PAV selection analysis between CL-cultivar and CL-landrace.

**Additional file 6: Table S5.** The result of PAV selection analysis between CL-landrace and CM.

**Additional file 7: Table S6.** The raw gene names and new gene names.

## Data Availability

The watermelon pangenome assembly and annotation are available at Figshare. database (https://figshare.com/articles/dataset/watermelon_pan-genome/19085471). WGS data were downloaded form NCBI Sequence Read Archive (SRA) through the SRA accession numbers SRP188834 (https://www.ncbi.nlm.nih.gov/sra/?term=SRP188834%20).
