# Supplementary material for: Pan-genome of Citrullus genus highlights the extent of presence/absence variation during domestication and selection
Source: BMC Genomics. 2023 Jun 15;24:332. doi: 10.1186/s12864-023-09443-w (PMC10273549; doi:10.1186/s12864-023-09443-w)
Supplement: Supplementary file 1 — Additional file 1: Figure S1. Heatmap of NBS (A), RLK (B), RLP (C), TM-CC (D) and all RGAs (E) presence and absence variations in different accessions. [file 12864_2023_9443_MOESM1_ESM.docx]

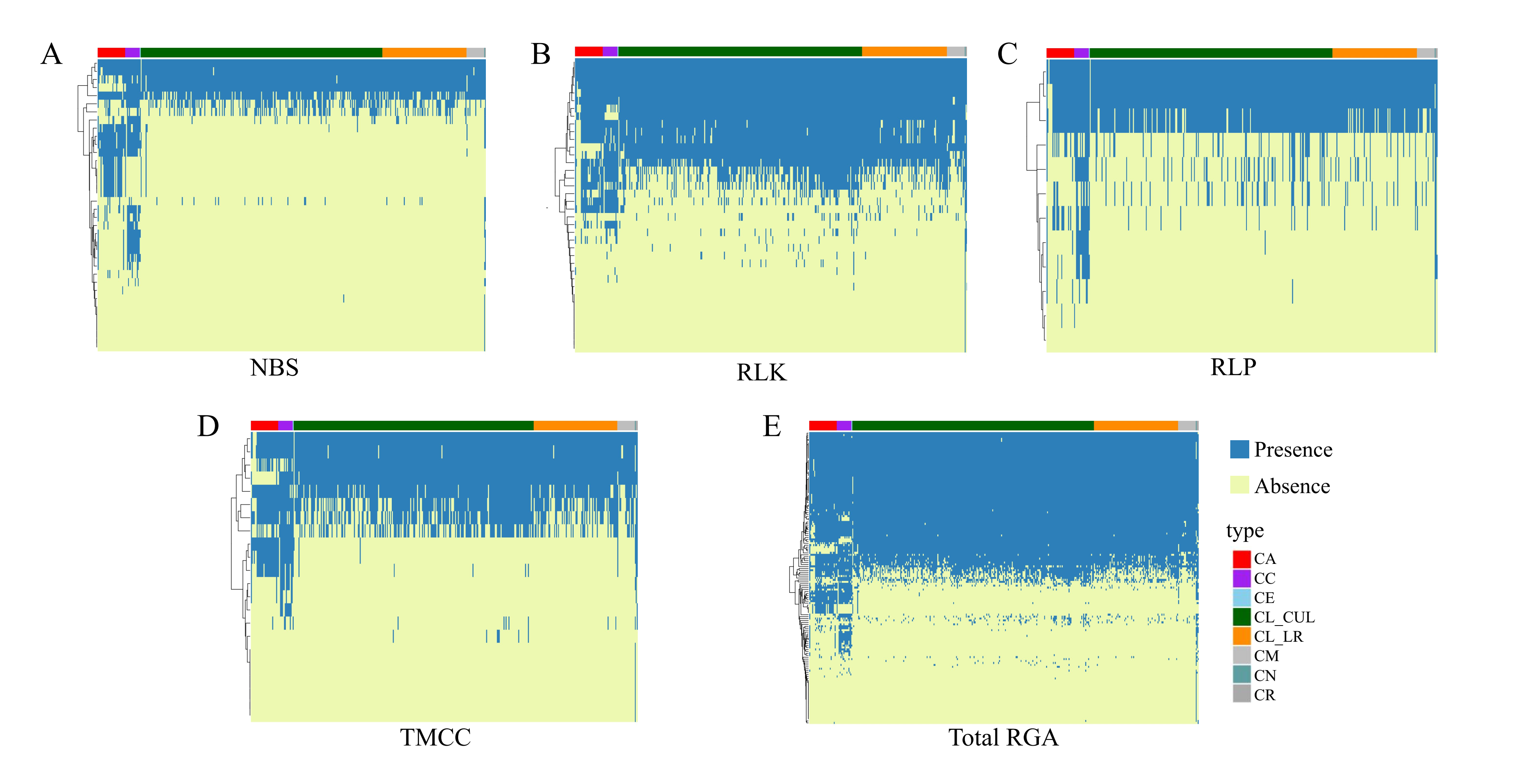


Figure S1 Heatmap of NBS (A), RLK (B), RLP (C), TM-CC (D) and all RGAs (E) presence and absence variations in different accessions.

Table S1: List of samples used for Citrullus genus pan-genome construction.

Table S2: List of the SRA number of RNA-seq data used for gene structure annotation.

Table S3: The result of PAV selection analysis between CL-cultivar and CM.

Table S4: The result of PAV selection analysis between CL-cultivar and CL-landrace.

Table S5: The result of PAV selection analysis between CL-landrace and CM.

Table S6: The raw gene names and new gene names.
